# Assessment of perioperative stress in colorectal cancer by use of *in vitro* cell models: a systematic review

**DOI:** 10.7717/peerj.4033

**Published:** 2017-11-17

**Authors:** Tove Kirkegaard, Mikail Gögenur, Ismail Gögenur

**Affiliations:** Center for Surgical Science, Department of Surgery, Zealand University Hospital, Koege, Denmark

**Keywords:** Proliferation, Colorectal cancer, Invasion, Migration, Surgery, *In vitro* models, Apoptosis

## Abstract

**Background:**

The perioperative period is important for patient outcome. Colorectal cancer surgery can lead to metastatic disease due to release of disseminated tumor cells and the induction of surgical stress response. To explore the overall effects on surgically-induced changes in serum composition, *in vitro* model systems are useful.

**Methods:**

A systematic search in PubMed and EMBASE was performed to identify studies describing *in vitro* models used to investigate cancer cell growth/proliferation, cell migration, cell invasion and cell death of serum taken pre- and postoperatively from patients undergoing colorectal tumor resection.

**Results:**

Two authors (MG and TK) independently reviewed 984 studies and identified five studies, which fulfilled the inclusion criteria. Disagreements were solved by discussion. All studies investigated cell proliferation and cell invasion, whereas three studies investigated cell migration, and only one study investigated cell death/apoptosis. One study investigated postoperative peritoneal infection due to anastomotic leak, one study investigated mode of anesthesia (general anesthesia with volatile or intravenous anesthetics), and one study investigated preoperative intervention with granulocyte macrophage colony stimulating factor (GMCSF). In all studies an increased proliferation, cell migration and invasion was demonstrated after surgery. Anesthetics with propofol and intervention with GMCSF significantly reduced postoperative cell proliferation, whereas peritoneal infection enhanced the invasive capability of tumor cells.

**Conclusion:**

This study suggests that *in vitro* cell models are useful and reliable tools to explore the effect of surgery on colorectal cancer cell proliferation and metastatic ability. The models should therefore be considered as additional tests to investigate the effects of perioperative interventions.

## Introduction

Tumor resection combined with radio/chemotherapy is the mainstay for treatment of colorectal cancer. However, despite expected curative surgical tumor resection, the recurrence rate is high, and one third of the patients with colorectal cancer will experience residual disease (Danish Colorectal Cancer Database, 2016). It is well known that tumor resection can induce surgical stress response, and also cause the release of disseminated tumor cells into the circulation. Surgical stress response is characterized by immune suppression, systemic inflammatory response, and excess reactive oxygen species (Søndergaard & Gögenur, 2015; McMillan, Canna & McArdle, 2003; Roxburgh et al., 2009), leading to favorable conditions for the remaining cancer cells to grow, and, consequently, increase the risk of getting residual disease (Van der Bij et al., 2009).

The perioperative period is short but important for cancer outcome. In particular the first months of the postoperative period are critical (Van der Bij et al., 2009). Genetic changes of blood components occur rapidly within the first 4 to 12 h postoperatively, and their expression levels remain high for the first days and up to several weeks after surgery (Xiao et al., 2011). The metastatic process is complex, and includes several biological processes such as cell detachment, expression and release of proteolytic enzymes, which are able to degrade extracellular matrix, induce cell migration, and cell invasion into distant organs consequently leading to distant cancer recurrence (Bird, Mangnall & Majeed, 2006). For understanding of the process leading to cancer recurrence, it is crucial to exploring the molecular mechanisms activated due to surgery. Previously, much focus has been on identifying single plasma components, and exploring individual mechanisms leading to development of metastatic disease. However, it is now well-recognized that cellular mechanisms, by which tumor recurrence is enhanced postoperatively, are multifactorial processes, involving several plasma molecules and a network of different mechanisms (Xiao et al., 2011). To explore the overall effects of surgically-induced changes of plasma proteins on postoperative tumor growth, *in vitro* tumor cell models can be used. Moreover, they can be used to investigate different types of surgery, anesthetics, and preoperative interventions. Our aim was therefore to systematically review the literature concerning the use of *in vitro* models to investigate cancer cell growth and metastatic ability of serum taken pre- and postoperatively from patients undergoing surgery for colorectal cancer.

## Materials and Methods

This systematic review was performed according to the PRISMA (Preferred Reporting Items for Systematic Reviews and Meta-analyses) guidelines (Moher et al., 2009). The selection of papers for the study was based on the PICO principles (Moher et al., 2009). The population (P) of interest was patients diagnosed with colon or rectal cancer. The intervention (I) was surgery for colon or rectal cancer, both minimally invasive and open conventional surgery. The comparison (C) was at least one blood sample taken pre- and postoperatively. The outcome (O) was the use of serum samples to investigate cell growth/proliferation, cell migration, cell invasion and/or cell death/apoptosis in *in vitro* cell models. The detailed systematic literature search was conducted in PubMed and EMBASE with no start date and April 2016 as the end date. The full PubMed and EMBASE search strategy is reported in Appendix S1. No review protocol exists. For study selection, Covidence online software (http://www.covidence.org) was used. Two reviewers (MG and TK) independently reviewed title and abstracts for all identified studies following full text assessment of eligible studies. From these full text citations, those that satisfied all criteria for study inclusion were included in the review. Discrepancies were discussed and resolved by consensus between MG and TK. To identify additional studies for the systematic review, all relevant references from identified papers were reviewed by MG and TK.

For bias assessment, the Newcastle-Ottawa quality assessment scheme, which is a “star-based” scoring system, was modified. The scheme was modified in accordance with the identified bias in the three categories: selection, compatibility and outcome. In the selection category, we specifically looked for the patient population as a true representative of the population, if the patients have received neoadjuvant chemotherapy, if there were differences between the treatment groups (if more than one treatment group was explored), if blood samples were collected pre-and postoperatively, and if the study used *in vitro* studies. In the compatibility category, comparison between the cohorts based on design and analysis, e.g., if the patient cohort was homogeneous, how the *in vitro* data were collected and if cancer cells were used as the *in vitro* model system was investigated. In the outcome category, the recording of the study results, were they properly recorded, and was the follow-up long enough to detect the outcome were studied.

## Results

### Study selection

**Figure 1:**
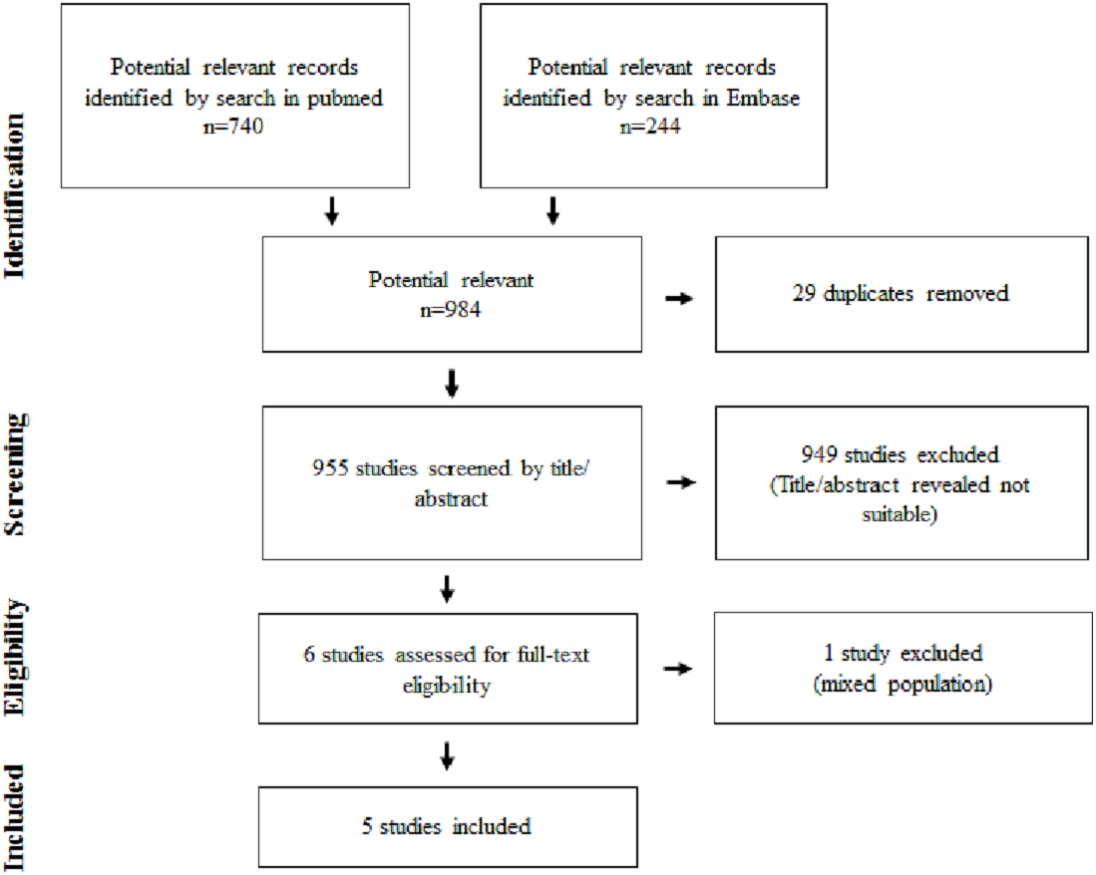
Flow chart of study selection.

**Figure 2:**
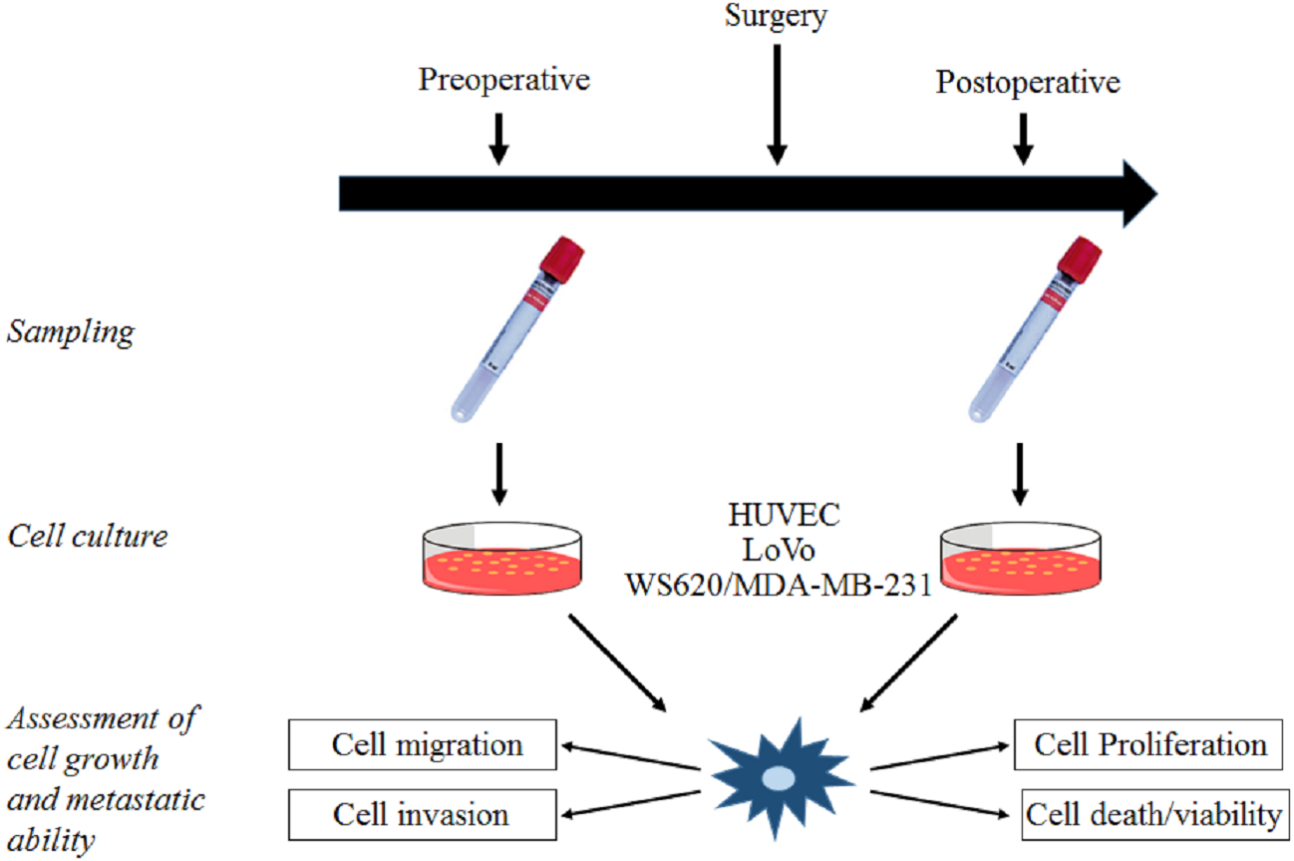
Study flow. Illustration of the study flow from blood sampling and *in vitro* cultures to assessment of cell growth and metastatic ability. HUVEC, human umbilical vein endothelial cells.

In total, 984 abstracts, 740 abstracts from PubMed and 244 abstracts from EMBASE were identified (Fig. 1). When 29 duplicates were excluded and title/abstracts reviewed in the remaining 955 abstracts, 949 abstracts were excluded. The remaining six papers were reviewed in details. Of these, five papers were included in the study (Fig. 2) (Kumara et al., 2009; Shantha Kumara et al., 2012; Salvans et al., 2014; Xu et al., 2016; Shantha Kumara et al., 2009). The last study was excluded as the patient group consisted of patients undergoing surgery for either colorectal cancer or gastric bypass and was therefore not a homogeneous group of patients undergoing surgery for colorectal cancer (Kirman et al., 2002). No additional studies were identified after reviewing references in the five included papers. For further analysis, we divided the studies into studies investigating cell proliferation/cell growth, cell migration, cell invasion and apoptosis/cell death (Tables 1 and 2). All five studies investigated cell proliferation and cell invasion, whereas three studies investigated cell migration (Kumara et al., 2009; Shantha Kumara et al., 2012; Salvans et al., 2014). Only one study investigated cell death/apoptosis (Xu et al., 2016). Two studies used cancer cell lines (SW620, derived from Human Caucasian colon adenocarcinoma; MDA-MB-231, derived from invasive ductal carcinoma and LoVo, derived from colorectal adenomacarcinoma) in their *in vitro* studies (Salvans et al., 2014; Xu et al., 2016), whereas the remaining three studies used human endothelial vein endothelial cell (HUVEC; derived from the endothelium of veins from the umbilical cord) (Kumara et al., 2009; Shantha Kumara et al., 2012; Shantha Kumara et al., 2009). In two studies, minimal invasive colorectal surgery was performed (Kumara et al., 2009; Shantha Kumara et al., 2009). Open conventional surgery was performed in the remaining three studies (Shantha Kumara et al., 2012; Salvans et al., 2014; Xu et al., 2016). One study investigated postoperative peritoneal infection due to anastomotic leak (Salvans et al., 2014), one study investigated general anesthesia with gas or propofol (Xu et al., 2016), and one study investigated preoperative intervention with GMCSF (Shantha Kumara et al., 2009).

**Table 1:** Study characteristics.

First author	Journal	No of patients	Intervention	Comparator	Cell lines
Kumara et al. (2009)	Annals of surgery	105	Minimal invasive colorectal resection	Pre-operative vs POD^a^7 and at varying times for up to 2 months.	Human umbilical vein endothelial cell (HUVEC)
Shantha Kumara et al. (2012)	Surgical endoscopy	53	Open surgery for rectal (*n* = 25) or colon (*n* = 28) cancer	Pre-operative vs POD7-33	Human Umbilical Vein Endothelial cell (HUVEC)
Salvans et al. (2014)	Annals of Surgery	94	Colorectal cancer surgery with (*n* = 47) or without (*n* = 47) infection from anastomotic leak (*n* = 34) or interabdominal abscess (*n* = 13)	Pre-operative vs POD4	Colon cancer cell lines: WS620 (invasion assay) Breast cancer cell lines: MDA-MB-231 (proliferation and migration)
Xu et al. (2016)	Anaesthesia	40	Open surgery for colon cancer, receiving general anesthesia with gas (*n* = 20) or propofol (*n* = 20)	Pre-operative vs POD1	Colon cancer cell line: LoVo
Shantha Kumara et al. (2009)	EJSO	59	Minimal invasive colorectal resection and intervention with GMCSF (*n* = 29) or placebo (*n* = 30)	Pre-operative vs POD5	Human umbilical vein endothelial cell (HUVEC)

**Notes.**

a POD: postoperative day.

**Table 2:** Outcome/results from the studies.

Author	Measurement/methods	Outcome/results			
		Cell proliferation (vs PreOP^d^ samples)	Cell migration (vs PreOP samples)	Cell invasion (vs PreOP samples)	Apoptosis (vs PreOP samples)
Kumara et al. (2009)	Cell proliferation: endothelial cell branch point formation assay	POD^c^7-13: increased (*p* = 0.0001)	POD7-13: increased (*p* = 0.001)	POD7-13: increased (*p* = 0.001)	NA^a^
	Cell migration: CBA 100 Cytoselect cell migration assay (Cell Biolabs Inc)	POD14-20: increased (*p* = 0.001)	POD14-20: increased (*p* = 0.001)	POD14-20: increased (*p* = 0.010)	
	Cell invasion: CBA 100 Cytoselect kit (Cell Biolabs Inc)	POD21-27: unchanged	POD21-27: unchanged	POD21-27: unchanged	
Shantha Kumara et al. (2012)	Cell proliferation: endothelial cell branch point formation assay	POD7-13: increased (*p* < 0.0001)	POD 7-13: increased (*p* < 0.0001)	POD 7-13: increased (*p* < 0.0001)	NA
	Cell migration: CBA 100 Cytoselect cell migration assay (Cell Biolabs Inc)	POD14-20: increased (*p* < 0.0001)	POD14-20: increased (*p* < 0.0001)	POD14-20: increased (*p* < 0.0001)	
	Cell invasion: CBA 100 Cytoselect kit (Cell Biolabs Inc)	POD21-33: unchanged (*p* = 0.09)	POD21-33: unchanged	POD21-33: increased (*p* = 0.04)	
		OS vs MICS: unchanged	OS vs MICS: sign week 3	OS vs MICS: sign week 2
Salvans et al. (2014)	Cell proliferation: colorimetric assay (Landegren and Givens)	POD4 (infection): increased (*p* = 0.013)	POD4 (infection): increased (*p* < 0.05)	POD4 (infection): unchanged	NA
	Cell migration: Boyden chamber assay (Corning Life Sceinces)				
	Cell invasion: Boyden chamber assay (Corning Life Sceinces)				
Xu et al. (2016)	Cell proliferation: MMT assay (Sigma)	POD1: gas vs. propofol: reduced (*p* = 0.005)	NA	POD1: PEA vs SGA: reduced (*p* < 0.001)	Apoptosis: POD1: gas vs. propofol: increased (*p* < 0.001)
	Cell invasion: Boyden chamber assay (BD Biosciences)				
	Cell viability/apoptosis: ApoLive-Glo Multiplex assay (promega)				
Shantha Kumara et al. (2009)	Cell proliferation: Endothelial cell branch point formation by ECM625 angiogenesis kit (Chemicon)	POD5 (control): increased (*p* = 0.001)	NA	POD5 (control): unchanged	NA
	Cell invasion: CBA 100 Cytoselect kit (Cell Biolabs Inc.)	POD5 (GMCSF): reduced (*p* = NS^b^)		POD5 (GMCSF): reduced (*P* = NS)	

**Notes.**

a NA: not assessed.

b NS: not significant.

c POD: postoperative day.

d PreOP: preoperative.

### Risk of bias

**Figure 3:**
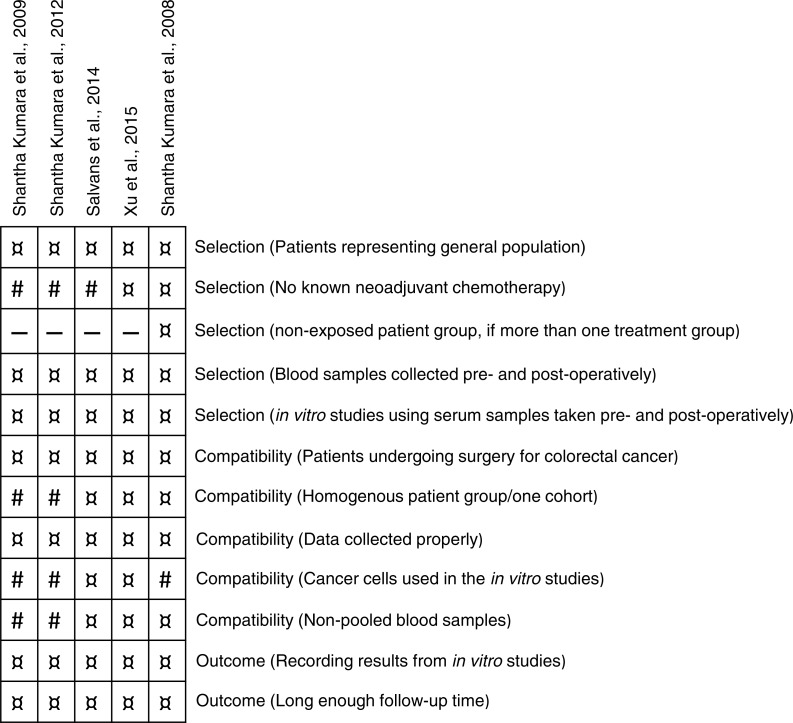
Bias assessment. Bias assessment using a modified Newcastle-Ottawa scale. The studies were assessed on three perspectives: selection of the study groups, comparability of the groups and if the studies met the outcome. ¤, Indicated bias item was met in the study. #: Indicated bias item was not met in the study.

Study bias was assessed using a modified Newcastle-Ottawa quality assessment scheme (Higgins & Green, 2011) defined in sections of selection, compatibility and outcome. The maximum score was 11 stars (12 if the study was a randomized trial with a control group) and the overall assessment scores varied from 7–11 (Fig. 3). In the selection section, patients from three studies had either received neoadjuvant chemotherapy or no statement was given (Kumara et al., 2009; Shantha Kumara et al., 2012; Salvans et al., 2014). In the compatibility section, colon cancer cell lines were only used in two studies (Salvans et al., 2014; Xu et al., 2016). Two study populations consisted of two patient cohorts (Kumara et al., 2009; Shantha Kumara et al., 2012), one study was a prospective matched cohort study (Salvans et al., 2014) and two studies were randomized clinical studies (Xu et al., 2016; Shantha Kumara et al., 2009). In two studies, blood samples were pooled into time-periods of seven days (Kumara et al., 2009; Shantha Kumara et al., 2012).

### Cell proliferation

Cell proliferation was measured in all five included studies, but with different assays. Cell proliferation was measured by determination of endothelial cell branch point formation of HUVECs (Kumara et al., 2009; Shantha Kumara et al., 2012; Shantha Kumara et al., 2009), or cancer cell proliferation measured by colorimetric assays (Salvans et al., 2014; Xu et al., 2016). Due to lack of samples at specific days, postoperative blood samples were pooled in time-periods of seven days in two studies (Kumara et al., 2009; Shantha Kumara et al., 2012). Compared with preoperative serum, postoperative serum stimulated *in vitro* endothelial cell branch point formation two and three weeks after minimal invasive (*p* = 0.0001 and *p* = 0.010, respectively) and open (*p* = 0.0001 and *p* = 0.0001, respectively) surgery for colorectal tumor resection (Kumara et al., 2009; Shantha Kumara et al., 2012). The effect was seen from postoperative day five (Shantha Kumara et al., 2009) and until three weeks after surgery (Kumara et al., 2009; Shantha Kumara et al., 2012). Serum taken four weeks after surgery did not significantly increase endothelial cell branch point formation compared with the effect of preoperative serum (Kumara et al., 2009; Shantha Kumara et al., 2012). No differences in endothelial cell branch point formation were seen postoperatively when comparing minimal invasive and open surgery (Kumara et al., 2009; Shantha Kumara et al., 2012). When using colorimetric assay, serum from postoperative day one and four also significantly stimulated cancer cell proliferation compared with serum taken preoperatively (*p* = 0.005 and *p* = 0013, respectively) (Salvans et al., 2014; Xu et al., 2016). Taken together, all five studies showed that postoperative serum stimulated *in vitro* cell proliferation from 24 h and up to three weeks after minimal invasive or open conventional surgery for colorectal cancer.

### Cell migration

Cell migration was investigated in three of the five included studies (Kumara et al., 2009; Shantha Kumara et al., 2012; Salvans et al., 2014). Compared to serum taken preoperatively, endothelial cell migration was significantly increased when stimulated with serum taken two and three weeks postoperatively, irrespective of patients undergoing minimal invasive (*p* = 0.001) or open conventional (*p* < 0.0001 and *p* = 0.0001 for week 2 and 3, respectively) surgery (Kumara et al., 2009; Shantha Kumara et al., 2012). Moreover, migration of HUVECs was significantly higher when cultured with serum taken three weeks after open conventional surgery, compared with serum from minimal invasive surgery (*p* < 0.001) (Salvans et al., 2014; Xu et al., 2016). Serum from patients with peritoneal infection after surgery significantly increased cell migration compared with controls (*p* < 0.05) (Salvans et al., 2014). Collectively, these results show that postoperative serum taken from four days and up to three weeks after surgery, significantly stimulated *in vitro* cell migration of both endothelial cells and cancer cells, and that open conventional surgery tend to improve cell migration compared to minimal invasive surgery.

### Cell invasion

Cell invasion, as measured by invasion of cells through a porous membrane of the extracellular matrix, was determined in all five included studies (Kumara et al., 2009; Shantha Kumara et al., 2012; Salvans et al., 2014; Xu et al., 2016; Shantha Kumara et al., 2009). In two studies, postoperative blood samples were combined into seven-days’ time periods. In these studies, cell invasion was increased up to three weeks after surgery compared with samples taking preoperatively, regardless which surgical method was performed (Kumara et al., 2009; Shantha Kumara et al., 2012). For patients undergoing open tumor resection, blood samples from postoperative day 21–33 (week four) also significantly stimulated invasion of HUVECs compared to preoperative serum samples (*p* = 0.04). Moreover, invasion of HUVECs was significantly higher when cultured with serum taken two weeks after open conventional surgery, compared with serum from minimal invasive surgery (*p* = 0.036) (Salvans et al., 2014; Xu et al., 2016). Serum from patients with peritoneal infection did not increase postoperative cell invasion (Salvans et al., 2014), however, serum from patients receiving anesthesia with propofol inhibited cancer cell invasion on postoperative day one compared with general anesthesia with gas (*p* = 0.004) (Xu et al., 2016). Preoperative intervention with GMCSF significantly reduced endothelial cell invasion at postoperative day five compared with preoperative taken serum (Shantha Kumara et al., 2009). Collectively, these results show that postoperative serum taken from four days and up to four weeks after surgery significantly stimulated *in vitro* cell invasion of endothelial cells and cancer cells regardless of the surgical method. Anesthesia with propofol and preoperative treatment with GMCSF prevented postoperative cancer cell invasion.

### Cell viability/apoptosis

Only one study investigated cell death/apoptosis (Xu et al., 2016) and showed that the viability of cancer cells cultured in the presence of postoperative serum taken from patients, who had received general anesthesia with gas, was higher compared with serum from patients receiving anesthesia with propofol (*p* = 0.01). Similarly, apoptosis was increased in serum from patients receiving anesthesia with propofol compared to patients receiving general anesthesia with gas (*p* < 0.001). Collectively, this shows that, compared with general anesthesia with gas, anesthesia with propofol reduced postoperative cell viability and increased cell death in cancer cells.

### Plasma protein measurements in combination with *in vitro* models

In two of the included papers, plasma protein levels were determined in parallel to the *in vitro* studies (Kumara et al., 2009; Shantha Kumara et al., 2009). Changes in pro-angiogenic proteins: vascular endothelial growth factor (VEGF), Angiopeotin-1 and -2 in serum samples collected preoperatively and during the first two postoperative months, were determined by enzyme-linked immunosorbent assay (ELISA). Expression of VEGF was significantly increased at postoperative day five, seven, and 13. The expression of angiopoitin-2 was increased in all collected postoperative serum samples. No significant changes were seen in the expression of angiopoitin-1 (Kumara et al., 2009). Compared to preoperative taken serum, the expression of soluble Vascular Endothelial Growth Factor Receptor 1 (sVEGFR1) was increased at postoperative day one and five in both placebo and GMCSF-treated group (Shantha Kumara et al., 2009), but the level was higher in the GMCSF-treated group. Angiopoitin-2 expression was increased in postoperative samples from both groups, but more pronounced in the GMCSF-group at postoperative day five. Expression of angiopoitin-1 was unchanged in both groups at any time point. The level of VEGF was significantly increased in both placebo and GMCSF-treated group at postoperative day five. None of the studies made correlations between the level of specific perioperative plasma proteins and the results from the *in vitro* models.

## Discussion

We identified five studies using *in vitro* models to compare the level of cell proliferation/cell growth, cell migration, cell invasion and cell viability/apoptosis in serum samples taken pre- and postoperatively. All five studies demonstrated that surgery-induced changes in plasma components lead to changes in cell growth and metastatic ability of both endothelial and cancer cells. Regardless of the surgical method and if endothelial or cancer cells were used, postoperative *in vitro* cell proliferation was stimulated from 24 h and up to three weeks after surgery, whereas *in vitro* cell migration and invasion were stimulated from four days and up to three weeks after surgery. Finally, anesthesia with propofol and preoperative treatment with GMCSF prevented postoperative cancer cell invasion.

A modified Newcastle-Ottawa scheme was used to identify bias. HUVECs have become the standard for many cell-based assays, and in three of the five studies HUVECs were used instead of cancer cells in the *in vitro* models (Kumara et al., 2009; Shantha Kumara et al., 2012; Shantha Kumara et al., 2009). A strength in the review was that similar outcomes have been measured in all five included studies. Although different cell lines were used, the results from the *in vitro* models were similar among the included studies. This confirms that *in vitro* models are reliable and useful in different settings. In contrast, a limitation in the review was that different cell lines were used and that there were variations among the studies in the days investigated postoperatively. In three studies, blood samples were investigated at day one, four or five postoperatively (Salvans et al., 2014; Xu et al., 2016; Shantha Kumara et al., 2009) whereas in the remaining two studies blood samples from different days were pooled into time-periods of seven days (Kumara et al., 2009; Shantha Kumara et al., 2012). Although the results from the two types of blood sampling were similar, there was a risk of losing information when blood samples from several days were pooled. In the five included studies, the samples size was relative small, varied from 40–105 patients. However, as all studies showed similar stimulation of postoperative *in vitro* cell proliferation, cell migration and invasion, it indicates sufficient number of patients in all studies. The smallest study, including only 40 patients (20 in each arm), showed that anesthesia with propofol prevented postoperative cancer cell invasion (Xu et al., 2016), which is in line with the literature showing that propofol improves long-term cancer outcome compared to the use of general anesthesia with gas (Heaney & Buggy, 2012). Thus, the sample size seems sufficient in all included studies, but it will be preferred if larger confirming studies will be performed to confirm these results. The study population varied among the studies. One study consisted of patients from a prospective matched cohort study (Salvans et al., 2014), another was a combination of different prospective studies (Kumara et al., 2009). One study included patients from two plasma banks (Shantha Kumara et al., 2012), and two studies were randomized trials (Xu et al., 2016; Shantha Kumara et al., 2009). Thus, the heterogeneity of the study design is a limitation, which could make the studies difficult to compare. However, it is also a strength that irrespective of study designs, cell proliferation and metastatic ability was improved post-operatively. It is also a limitation that only one study investigated perioperative intervention and that not all studies mentioned if the patients had received neoadjuvant chemotherapy. Finally, three of the five studies were from the same research group (Kumara et al., 2009; Shantha Kumara et al., 2012; Shantha Kumara et al., 2009), however, although overlapping studies in two papers (Kumara et al., 2009; Shantha Kumara et al., 2012), only 13 patients receiving granulocyte macrophage colony stimulating factor (GMCSF) might have been included in two studies (Kumara et al., 2009; Shantha Kumara et al., 2009). We have, without success, tried to get information on these patients from the corresponding author on the paper.

Despite conflicting results regarding the reduced risk of disease recurrence among colorectal cancer patients when using minimal invasive surgery (Lacy et al., 2002; Kirman et al., 2005; Schwenk et al., 2000; Belizon et al., 2006; Heaney & Buggy, 2012), it is now general practice for most surgical procedures with the advantage of less postoperative morbidity and less surgical trauma (Lacy et al., 2002; Veldkamp et al., 2005). Minimal invasive surgery is associated with less postoperative changes in blood composition (Kirman et al., 2005; Schwenk et al., 2000; Belizon et al., 2006). Lower postoperative levels of interleukin-6, C-reactive protein and VEGF was found in serum from patients undergoing minimal invasive surgery compared to patients undergoing open conventional surgery (Belizon et al., 2006; Schwenk et al., 2000), and a decrease in the level of the cancer cell growth inhibitory protein, and Insulin-like growth factor-binding protein 3 (IGFBP-3), was determined in postoperative serum from patients undergoing open conventional surgery compared to minimal invasive surgery (Kirman et al., 2005). In this review, serum taken postoperatively significantly stimulated *in vitro* cell invasion and migration compared with serum taken preoperatively regardless of the surgical method.

The use of anesthesia with propofol for primary cancer resection improved long-term cancer outcome compared to the use of general anesthesia with gas (Heaney & Buggy, 2012). Here we found that the *in vitro* models could be used to determine differences between the uses of anesthetics. Postoperative cell proliferation and invasion were reduced when colon cancer cells were treated with serum from patients, who had received anesthesia with propofol. In contrast, serum from patients, who had received anesthesia with gas, reduced cancer cell death/apoptosis. The differences seen in the *in vitro* studies likely reflect the differences in surgery-induced release of serum components as previously shown (Xu et al., 2014). As none of the included studies correlated the level of specific perioperative plasma proteins with the results from the *in vitro* models, such investigations are warranted.

Surgically-induced stress response is a major problem resulting in increased morbidity, mortality and delay of initiation of oncological treatment. Although the first month after tumor resection in theory should be an ideal time for initiation of the oncological treatment, it has traditionally been a “no-touch” period due to concerns of recovery (Horowitz et al., 2015). However, it is of interest to explore the use of preoperative immunomodulation to reduce the risk of getting surgery-induced disease recurrence, and models to verify the effect of immune modulation is necessary. One of the included studies investigated the effect of perioperative intervention with GMCSF (Shantha Kumara et al., 2009). GMCSF prevents tumor growth in a murine carcinoma model (Hill et al., 1996), and here GMCSF reduced postoperative *in vitro* cell growth and invasion (Shantha Kumara et al., 2009). Thus, based on the included studies, *in vitro* models can be used as a reliable test to verify the effect of preoperative intervention to optimize the immune system, and the use of such studies should therefore be further investigated, preferably in combination with determination of changes in serum components during the perioperative period.

## Conclusion

This systematic review has shown that *in vitro* models can be used to investigate the multifunctional mechanisms activated upon surgery leading to increased cell growth and metastatic ability both in the context of minimal invasive/open conventional surgery, use of anesthesia, and preoperative interventions. Although we were only able to identify five rather heterogeneous studies, we suggest that the use of *in vitro* studies should be further explored as a tool to investigate the effect of intervention studies designed to improve and reduce the length of the postoperative period before oncological treatment.

##  Supplemental Information

10.7717/peerj.4033/supp-1 Supplemental Information 1 PRISMA checklist

10.7717/peerj.4033/supp-2 Appendix S1 Screenshot from (A) PubMed and (B) EMBASE showing the full search strategy used in the study

10.7717/peerj.4033/supp-3 Supplemental Information 3 PRISMA flow diagram

10.7717/peerj.4033/supp-4 Supplemental Information 4 The rationale for conducting the systematic review and the contribution that the systematic review makes

## Additional Information and Declarations

### Data Availability

The following information was supplied regarding data availability: This paper is a systematic review and does not contain raw data/code.

## References

[ref-1] Belizon A, Balik E, Feingold DL, Bessler M, Arnell TD, Forde KA, Horst PK, Jain S, Cekic V, Kirman I, Whelan RL (2006). Major abdominal surgery increases plasma levels of vascular endothelial growth factor: open more so than minimally invasive methods. Annals of Surgery.

[ref-2] Bird NC, Mangnall D, Majeed AW (2006). Biology of colorectal liver metastases: a review. Journal of Surgical Oncology.

[ref-3] Danish Colorectal Cancer Database (2016). Yearly Report.

[ref-4] Heaney Á, Buggy DJ (2012). Can anaesthetic and analgesic techniques affect cancer recurrence or metastasis?. British Journal of Anaesthesia.

[ref-5] Higgins JPT, Green S (2011). Cochrane handbook for systematic reviews of interventions Version 5.1.0 [updated March 2011]. http://handbook.cochrane.org.

[ref-6] Hill ADK, Redmond HP, Naama HA, Bouchier-Hayes D (1996). Granulocyte-macrophage colony-stimulating factor inhibits tumor growth during the postoperative period. Surgery.

[ref-7] Horowitz M, Neeman E, Sharon E, Ben-Eliyahu S (2015). Exploiting the critical perioperative period to improve long-term cancer outcomes. Nature Reviews Clinical Oncology.

[ref-8] Kirman I, Cekic V, Poltaratskaia N, Asi Z, Bessler M, Huang EH, Forde KA, Whelan RL (2002). Plasma from patients undergoing major open surgery stimulates *in vitro* tumor growth: lower insulin-like growth factor binding protein 3 levels may, in part, account for this change. Surgery.

[ref-9] Kirman I, Cekic V, Poltoratskaia N, Sylla P, Jain S, Forde KA, Whelan RL (2005). Open surgery induces a dramatic decrease in circulating intact IGFBP-3 in patients with colorectal cancer not seen with laparoscopic surgery. Surgical Endoscopy and Other Interventional Techniques.

[ref-10] Kumara HMCS, Feingold D, Kalady M, Dujovny N, Senagore A, Hyman N, Cekic V, Whelan RL (2009). Colorectal resection is associated with persistent proangiogenic plasma protein changes: postoperative plasma stimulates *in vitro* endothelial cell growth, migration, and invasion. Annals of Surgery.

[ref-11] Lacy AM, García-Valdecasas JC, Delgado S, Castells A, Taurá P, Piqué JM, Visa J (2002). Laparoscopy-assisted colectomy versus open colectomy for treatment of non-metastatic colon cancer: a randomised trial. Lancet.

[ref-12] McMillan DC, Canna K, McArdle CS (2003). Systemic inflammatory response predicts survival following curative resection of colorectal cancer. British Journal of Surgery.

[ref-13] Moher D, Liberati A, Tetzlaff J, Altman DG, Prisma Grp (2009). Preferred reporting items for systematic reviews and meta-analyses: the PRISMA statement. Annals of Internal Medicine.

[ref-14] Roxburgh CSD, Campbell SD, Salmond JM, Horgan PG, Oien KA, McMillan DC (2009). The relationship between the local and systemic inflammatory responses and survival in patients undergoing curative surgery for colon and rectal cancers. Journal of Gastrointestinal Surgery.

[ref-15] Salvans S, Mayol X, Alonso S, Messeguer R, Pascual M, Mojal S, Grande L, Pera M (2014). Postoperative peritoneal infection enhances migration and invasion capacities of tumor cells *in vitro*: an insight into the association between anastomotic leak and recurrence after surgery for colorectal cancer. Annals of Surgery.

[ref-16] Schwenk WC, Mansmann JU, Böhm B, Müller JM (2000). Inflammatory response after laparoscopic and conventional colorectal resections—results of a prospective randomized trial. Langenbeck’s Archives of Surgery.

[ref-17] Shantha Kumara HMC, Kirchoff D, Naffouje S, Grieco M, Herath SAC, Dujovny N, Kalady MF, Hyman N, Njoh L, Whelan RL (2012). Plasma from the second and third weeks after open colorectal resection for cancer stimulates *in vitro* endothelial cell growth, migration, and invasion. Surgical Endoscopy.

[ref-18] Shantha Kumara HMC, Kirman I, Feingold D, Cekic V, Nasar A, Arnell T, Balik E, Hoffman A, Baxter R, Conte S, Whelan RL (2009). Perioperative GMCSF limits the proangiogenic plasma protein changes associated with colorectal cancer resection. European Journal of Surgical Oncology.

[ref-19] Søndergaard ES, Gögenur I (2015). Oxidative stress may cause metastatic disease in patients with colorectal cancer. Ugeskrift for Laeger.

[ref-20] Van der Bij GJ, Oosterling SJ, Beelen RHJ, Meijer S, Coffey JC, Van Egmond M (2009). The perioperative period is an underutilized window of therapeutic opportunity in patients with colorectal cancer. Annals of Surgery.

[ref-21] Veldkamp R, Kuhry E, Hop WCJ, Jeekel J, Kazemier G, Bonjer HJ, Haglind E, Påhlman L, Cuesta MA, Msika S, Morino M, Lacy AM, COlon cancer Laparoscopic or Open Resection Study Group (COLOR) (2005). Laparoscopic surgery versus open surgery for colon cancer: short-term outcomes of a randomised trial. The Lancet Oncology.

[ref-22] Xiao W, Mindrinos MN, Seok J, Cuschieri J, Cuenca AG, Gao H, Hayden DL, Hennessy L, Moore EE, Minei JP, Bankey PE, Johnson JL, Sperry J, Nathens AB, Billiar TR, West MA, Brownstein BH, Mason PH, Baker HV, Finnerty CC, Jeschke MG, López MC, Klein MB, Gamelli RL, Gibran NS, Arnoldo B, Xu W, Zhang Y, Calvano SE, McDonald-Smith GP, Schoenfeld DA, Storey JD, Cobb JP, Warren HS, Moldawer LL, Herndon DN, Lowry SF, Maier RV, Davis RW, Tompkins RG, Inflammation and Host Response to Injury Large-Scale Collaborative Research Program (2011). A genomic storm in critically injured humans. Journal of Experimetnal Medicine.

[ref-23] Xu YJ, Chen WK, Zhu Y, Wang SL, Miao CH (2014). Effect of thoracic epidural anaesthesia on serum vascular endothelial growth factor C and cytokines in patients undergoing anaesthesia and surgery for colon cancer. British Journal of Anaesthesia.

[ref-24] Xu YJ, Li SY, Cheng Q, Chen WK, Wang SL, Ren Y, Miao CH (2016). Effects of anaesthesia on proliferation, invasion and apoptosis of LoVo colon cancer cells *in vitro*. Anaesthesia.

