# Supplementary figures and images for: Assessment of perioperative stress in colorectal cancer by use of in vitro cell models: a systematic review
Source: PeerJ. 2017 Nov 17;5:e4033. doi: 10.7717/peerj.4033 (PMC5695245; doi:10.7717/peerj.4033)

A)


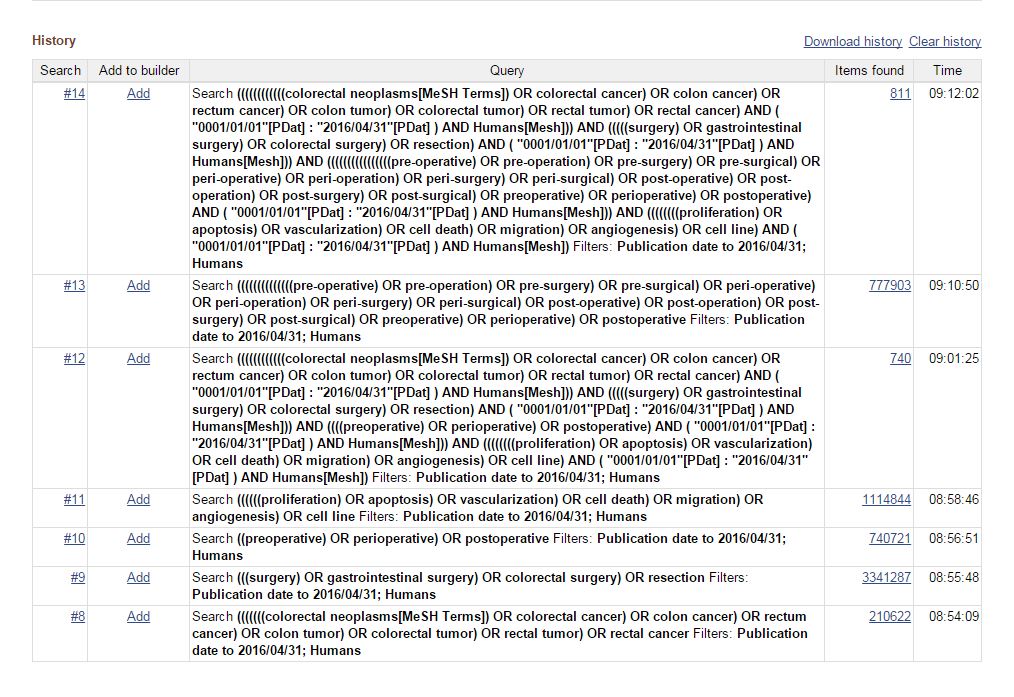


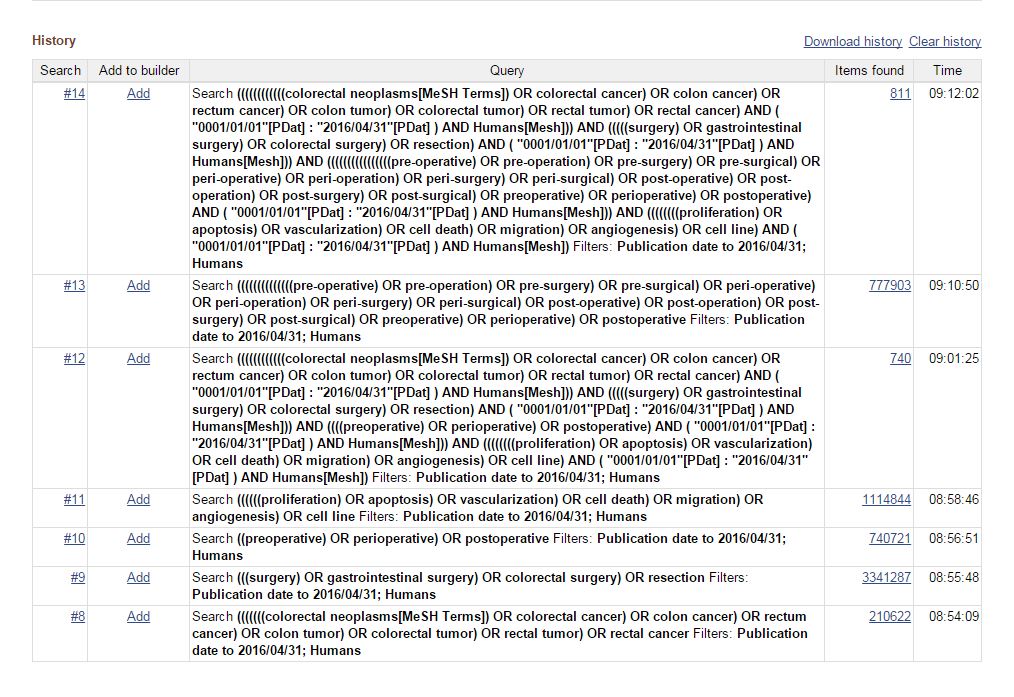


B)


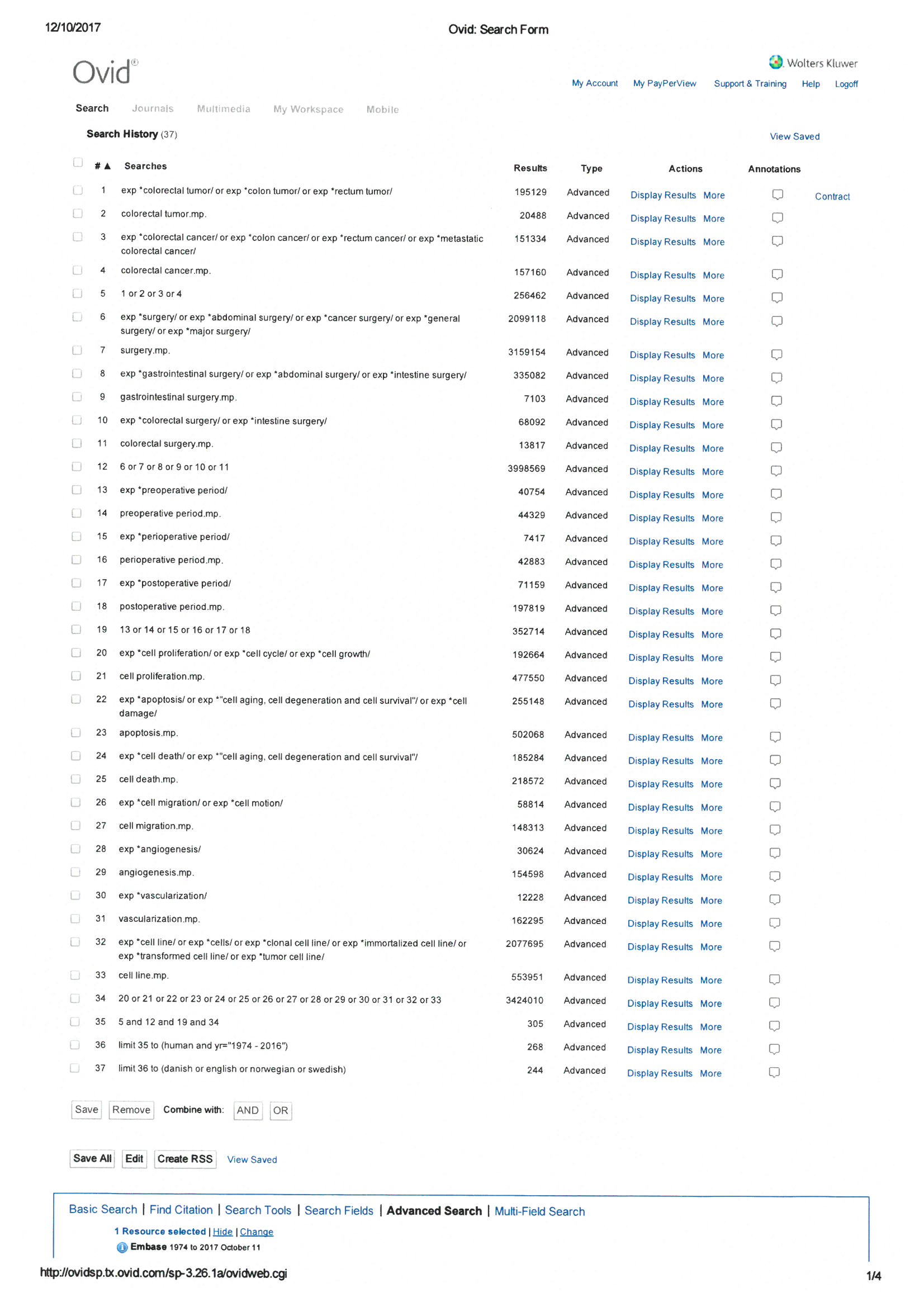

Supplement: Appendix S1 [file peerj-05-4033-s002.docx]
