# Supplementary material for: Assessment of perioperative stress in colorectal cancer by use of in vitro cell models: a systematic review
Source: PeerJ. 2017 Nov 17;5:e4033. doi: 10.7717/peerj.4033 (PMC5695245; doi:10.7717/peerj.4033)
Supplement: Supplemental Information 3 [file peerj-05-4033-s003.pdf]

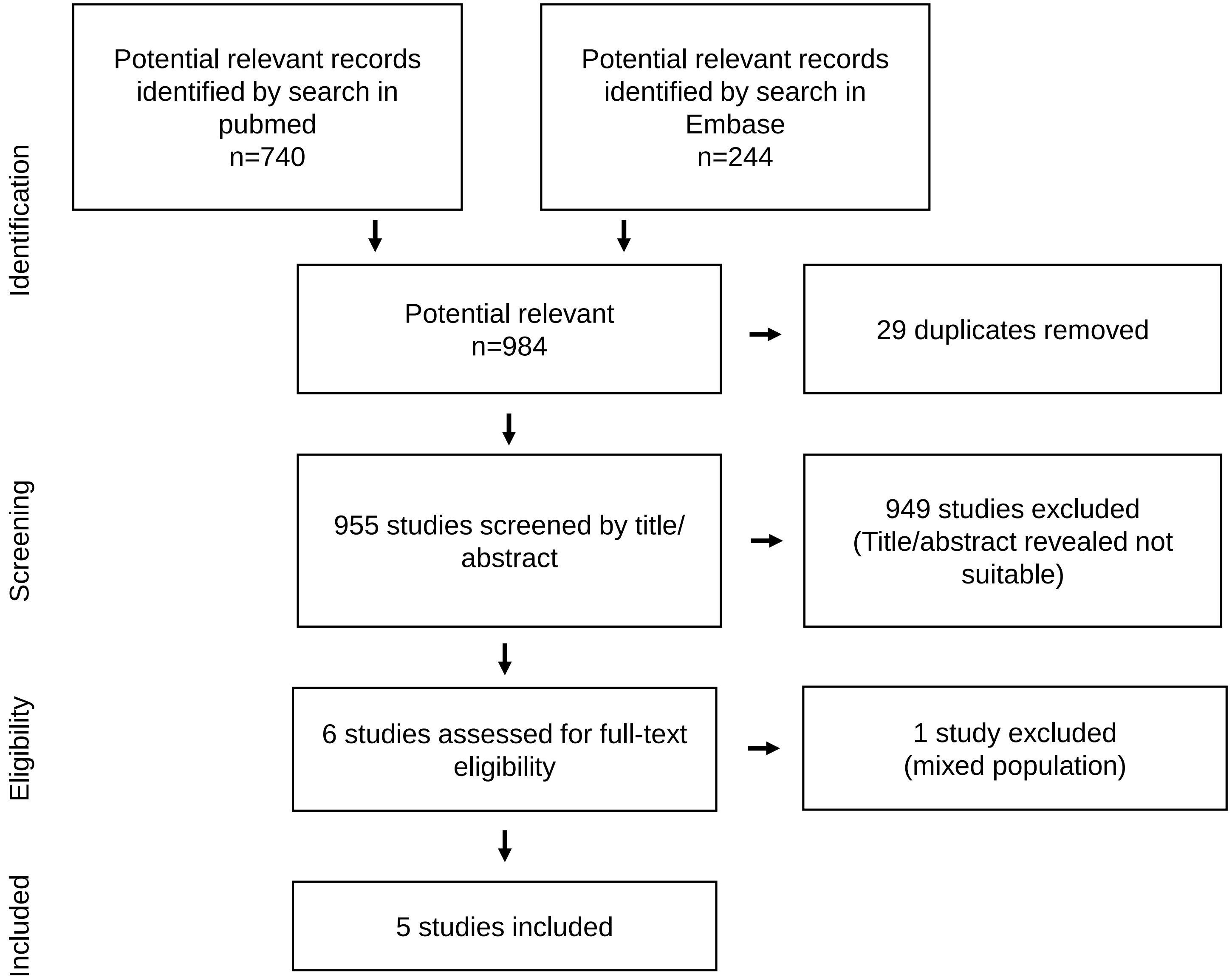
Identification
Potential relevant records identified by search in pubmed n=740
Potential relevant records identified by search in Embase n=244
Potential relevant n=984
29 duplicates removed
Screening
955 studies screened by title/ abstract
949 studies excluded (Title/abstract revealed not suitable)
Eligibility
6 studies assessed for full-text eligibility
1 study excluded (mixed population)
Included
5 studies included
