# Supplementary material for: Assessment of perioperative stress in colorectal cancer by use of in vitro cell models: a systematic review
Source: PeerJ. 2017 Nov 17;5:e4033. doi: 10.7717/peerj.4033 (PMC5695245; doi:10.7717/peerj.4033)
Supplement: Supplemental Information 4 [file peerj-05-4033-s004.docx]

1. **The rationale for conducting the systematic review:**

Even with the best surgical technique, colorectal cancer surgery can lead to metastatic disease. Surgical tumor resection can cause release of disseminated tumor cells and induce a stress response characterized by immune suppression and inflammatory response. Collectively, this can cause favorable conditions for the remaining cancer cells to grow and, consequently, increase the risk of getting residual disease. Our rationale for conducting the systematic review was to review the literature to find out if in vitro studies of cell lines cultured with serum obtained before and after surgery could give an indication for the ability of serum components to mediate increased cancer cell growth and metastatic ability.

**2. The contribution that the systematic review makes to knowledge in light of previously published related reports, including other systematic reviews**

We have not been able to identify other systematic reviews on this subject. However, in the five identified studies in this systematic review, we consistently found that *in vitro* models were reliable tools to explore multifunctional mechanisms activated upon colorectal surgery, leading to increased cell growth and metastatic ability. This was seen in the context of minimal invasive/open conventional colorectal surgery, use of anesthesia, and preoperative interventions. We therefore suggest that *in vitro* studies can be used to investigate and confirm the effect of intervention studies designed to improve and reduce the length of the postoperative period before oncological treatment, however, for implementation in clinical studies the models should be further explored.
